# Image-conditioned latent rectified flow models for 3D medical anomaly localisation

**DOI:** 10.3389/fradi.2026.1847193

**Published:** 2026-07-24

**Authors:** Matthew Baugh, Johanna P. Müller, Sarah Cechnicka, Kexin Gu Baugh, John Bonnici, James Myles, Bernhard Kainz

**Affiliations:** 1 Biomedical Image Analysis Group, Department of Computing, Imperial College London, London, United Kingdom; 2 IDEA Lab, Department of Artificial Intelligence in Biomedical Engineering, Friedrich-Alexander Universität Erlangen-Nürnberg, Erlangen, Germany; 3 Structured and Probabilistic Intelligent Knowledge Engineering Group, Department of Computing, Imperial College London, London, United Kingdom

**Keywords:** anomaly detection, anomaly localisation, medical image analysis, pseudo-healthy restoration, rectified flow models, self-supervised learning

## Abstract

**Introduction:**

Reconstruction-based methods offer a promising solution for unsupervised anomaly detection in medical imaging tasks. These methods train generative models on healthy data alone and identify anomalies as deviations between an input image and its pseudo-healthy reconstruction. The downfall of these methods is their dependence on two assumptions that often fail in practice: that models cannot reproduce unseen pathologies, yet can faithfully reconstruct healthy tissue. A recent image-conditioned diffusion approach explicitly addresses these issues by training a model to restore synthetic anomalies inserted into healthy images. However, it operates in 2D pixel space, discarding inter-slice context and incurring high computational cost.

**Methods:**

We address both limitations by performing image-conditioned restoration in a 3D latent space using a pretrained VAE and rectified flow, capturing volumetric context whilst drastically reducing computational overhead. To mitigate false positives introduced by VAE compression, we propose using the restoration change which measures the difference between the pseudo-healthy latent restoration and the VAE reconstruction of the original, rather than the standard reconstruction error. We further experiment with applying the synthetic anomaly training task directly in latent space to improve sensitivity to low-contrast anomalies.

**Results:**

We perform extensive experiments across various medical imaging benchmarks, including brain MRI and the newly released AADD dataset, comparing against reconstruction-based, feature-modelling, attention-based and self-supervised anomaly detection methods. Our image-conditioned rectified flow models establish a new state-of-the-art, with an ensemble of models trained with pixel-space and latent-space anomalies yielding the strongest overall performance.

**Discussion:**

These results demonstrate how incorporating 3D context enables better anomaly detection performance whilst also being ∼10 times faster. The difference in performance between models trained using latent-space and pixel-space anomalies suggests that further broadening of the anomaly imputation process could continue to improve the robustness of these models. Such improvements are certainly necessary, as the AADD benchmark is far from being saturated. Code is available at https://github.com/matt-baugh/img-cond-latent-rflow-model-ad.

## 1 Introduction

Unsupervised Anomaly Detection (UAD) aims to model the distribution of normal data and identify anomalies as deviations from it. Only requiring data from the normal distribution makes it an attractive alternative to standard supervised learning, which requires data from all classes of interest. It is particularly well-suited to medical imaging, where normal scans are abundant but the variety of pathologies is wide and with a range of differential prevalences. UAD has the added benefit of producing pixel-level anomaly maps without needing segmentation maps for training, which would otherwise be very time-consuming to annotate. Most methods achieve this by training a generative model to reconstruct the healthy training data, and at test time a comparison between the input sample and its reconstruction indicates whether any potential anomalies lie; healthy samples are typically reconstructed faithfully but anomalous samples that differ from the training distribution will have reconstruction errors. Furthermore, these reconstructions offer a degree of interpretability; they present the user with a pseudo-healthy version of the image, allowing them to see what the anomalous regions of the image should look like.

However, a drawback of reconstruction-based methods is that they depend on two key assumptions: **(A1)** that models trained only on healthy data cannot reconstruct anomalous features unseen during training; and **(A2)** that they can accurately reconstruct images drawn from the normative distribution. Recently, both assumptions have been challenged. The nuanced differences between healthy and anomalous medical scans lead to models reconstructing unhealthy regions [1], requiring tuning of the model capacity to prevent them from being reconstructed [2]. On the other hand, healthy regions are also often imperfectly reconstructed due to sharp edges or their general complexity [3], resulting in false positives predicted across the image. These can be removed using postprocessing; however, knowledge of the size and type of test time anomalies is required to prevent the predictions for anomalous areas from being removed as well [4].

To avoid these shortcomings, Baugh et al. [5] trained a diffusion model to explicitly generate a pseudo-healthy restoration of an input image. They achieved this using image-conditioning; during training, a healthy image was used as the generation target while the same image with a synthetic anomaly inserted into it was provided as conditioning to the diffusion model. The diffusion model could then learn to identify different regions of the conditioning image, retaining healthy regions and correcting unhealthy ones. At test time, the diffusion model is conditioned on the input image and used to generate a pseudo-healthy version of it, which can be compared to the original image to produce an anomaly map.

**Figure 1:**
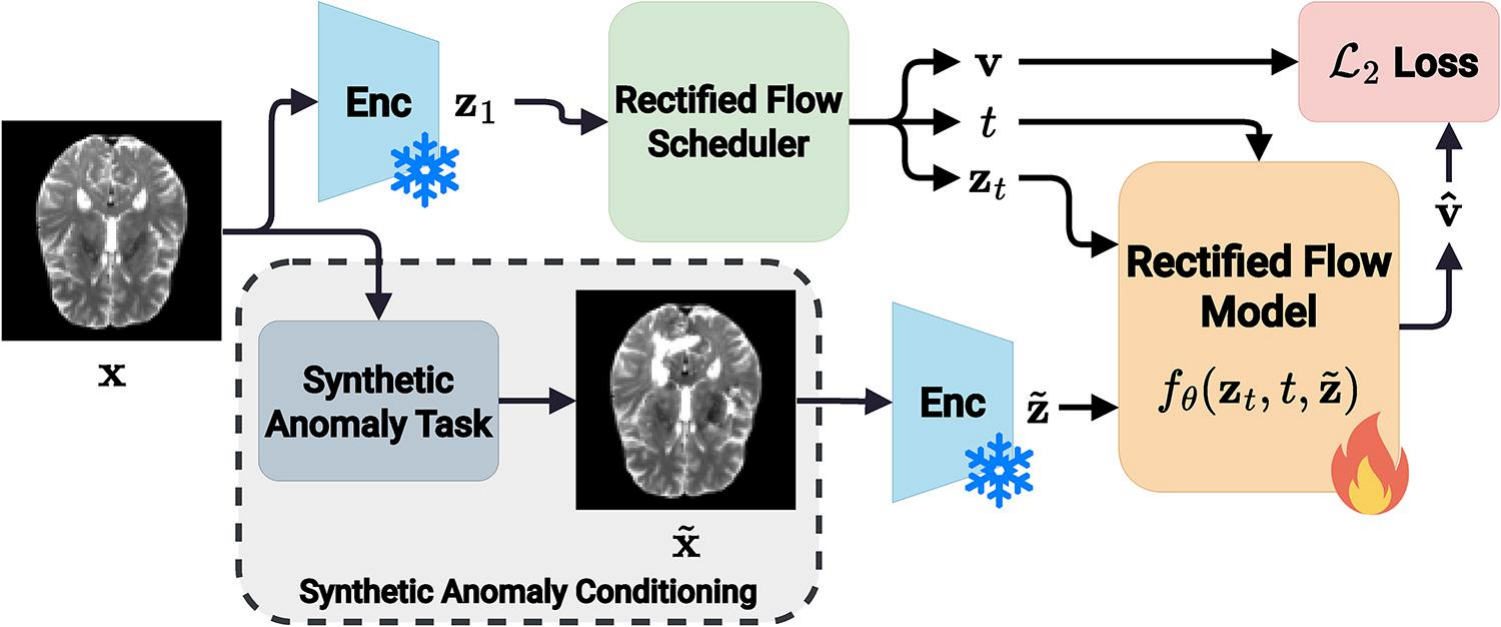
The training process of our model. We apply a synthetic anomaly task to the original image x to create a corrupted version $\tilde{x}$, and then encode both using the frozen MAISI VAE encoder [6] to create $z_{1}$ and $\tilde{z}$ respectively. The rectified flow scheduler then noises $z_{1}$ to make $z_{t}$, which is provided as input to the rectified flow model with $\tilde{z}$ and timestep t as conditioning. The model is trained to predict the velocity which corrects $z_{t}$ towards $z_{1}$ by leveraging the information in $\tilde{z}$.

Baugh et al. [5] achieved better performance through its anomaly-detection-centric training. However, this approach applied a 2D diffusion model independently to each axial slice. This lack of 3D context makes the normal distribution harder to learn as there is a high degree of structural variability across slices. For example, for an image showing small ventricles, the model may be uncertain whether they are small due to a pathology or because the slice is simply taken towards the top of the ventricle region. This is especially true for slices at the very top or very bottom of the brain, where very few pixels are visible, making it difficult to determine whether the content is normal. Secondly, the diffusion model operated in pixel space, meaning each denoising step occurred at a high resolution. This makes the process highly computationally expensive, and is the main limiting factor preventing the model from including 3D context. We improve upon this by making the following contributions:

1. We use a pretrained Variational Auto-Encoder (VAE) to perform image-conditioned restoration in a 3D latent space using rectified flow (Figure 1).
2. At inference time, we propose using the restoration change, comparing the pseudo-healthy restoration to the VAE reconstruction of the original, rather than the standard reconstruction error, in order to reduce the false positives introduced by the off-the-shelf VAE compression.
3. We experiment with applying the synthetic anomaly task within the latent space itself, reducing training time and achieving better performance for low-contrast anomalies.
4. We ensemble the models trained with pixel-space and latent-space anomalies, resulting in the best performance.

### 1.1 Related work

#### 1.1.1 Image reconstruction

The dominant paradigm in UAD trains a generative model on healthy data only, and computes a pixel-wise anomaly score as the residual between an input image and its reconstruction. Kingma and Welling [7] introduce the VAE, which has been widely adopted for this purpose, and Chen et al. [8] extend this with a restoration-based approach that iteratively updates the input image to match the learned normative prior rather than directly comparing reconstruction residuals. Ghorbel et al. [9] apply a hierarchical transformer autoencoder to the same reconstruction principle, exploiting the self-attention mechanism to better capture long-range spatial dependencies in brain MR images. GANs such as f-AnoGAN [10] have been explored in this setting, and Baur et al. [4] provide a systematic comparative evaluation of these approaches for brain MR imaging. A key limitation is that powerful decoders tend to reconstruct anomalous regions. MAISI [11] is a large-scale medical image synthesis framework that trains a VAE on large collections of medical images. Zhao et al. [6] extend their previous work with a rectified flow model.

Diffusion models have more recently been applied to the same reconstruction principle. Wolleb et al. [12] use the noising-denoising cycle of a Denoising Diffusion Probabilistic Model (DDPM), see Section 2.1 for more details, to translate pathological images towards healthy-looking counterparts, deriving an anomaly map from the difference. Pinaya et al. [13] apply the diffusion process in the latent space of a pretrained autoencoder, reducing inference time whilst retaining competitive performance. A limitation shared by these methods is that the reverse diffusion process can alter healthy tissue as well as pathological regions. Bercea et al. [14] aimed to tackle this by producing a coarse segmentation of confidently healthy tissue and use it as a mask to guide an inpainting network, so only suspected anomalous regions are resampled. Bercea et al. [15] took this idea further by integrating these intermediate healthy-tissue masks directly into the reverse diffusion process as implicit guidance, constraining edits to pathological regions whilst leaving healthy structure unchanged.

#### 1.1.2 Feature-modelling

Feature modelling methods pass inputs through a frozen pretrained encoder and model normality in the resulting embedding space rather than in pixel space. FAE [16] applies reconstruction-based scoring with the Structural Similarity Index Measure (SSIM) [17] to patch-level features from a pretrained network. PaDiM [18] fits a multivariate Gaussian to patch embeddings at each spatial position, and CFLOW-AD [19] uses conditional normalising flows to assign exact likelihoods to embeddings. Reverse distillation [20] detects anomalies from the discrepancy between a pretrained teacher encoder and a student decoder that reconstructs features in the forward direction. Lagogiannis et al. [21] benchmark these paradigms on multiple medical imaging datasets and show that feature modelling methods consistently outperform reconstruction-based baselines, and that domain-specific self-supervised pretraining further improves performance.

#### 1.1.3 Attention-based anomaly detection

Attention-based methods derive localisation maps from the gradients or activations of a trained model rather than from pixel-space residuals. ExpVAE [22] uses a GradCAM-style backpropagation procedure to extract per-latent-dimension attention maps from a one-class VAE, treating regions with atypical activations as anomalous. AMCons [23] uses inequality constraints to force the VAE’s attention maps to activate across the whole image during training on normal data, and introduces an entropy regulariser on the attention maps as an alternative to the KL regularisation, reducing the number of hyperparameters required.

#### 1.1.4 Self-supervised anomaly detection

Self-supervised methods generate synthetic local or global anomalies in normal data during training. Golan and El-Yaniv [24] train a classifier to distinguish between dozens of geometric transformations applied to normal images; at test time, the softmax statistics over the transformed versions of an input yield an anomaly score. This works well for semantic outliers but is less effective in medical imaging, where major anatomical structures are present regardless of pathology. Kascenas et al. [25] using Denoising autoencoders (DAEs) address this by adding spatially coarse noise to the input during training, so the model learns to erase anomalous structure rather than reproduce it. CutPaste [26] cuts a patch from a normal image and pastes it at a random location, then trains a classifier to detect such augmentations; the learned representations are sensitive to local structural irregularities. Tan et al. [27] first propose Foreign Image Interpolation (FPI), which linearly interpolated a patch from another sample into the input image and trained the model to predict the interpolation factor pixel-wise, giving both image-level and pixel-level anomaly scores and ranking first in the MOOD challenge 2020. This is extended in Poisson Patch Interpolation (PII) [28], which improved the interpolation strategy to use Poisson image blending, seamlessly integrating the foreign patch into the surrounding sample. NSA [29] further develops this line of work by blending patches of varying sizes from separate images into different locations using PII, producing a wider range of synthetic anomalies that more closely resemble natural sub-image irregularities. Müller et al. [30] build further on PII by introducing a confidence-aware training objective, with loosened feature locality constraints through local probabilistic blending (P-PII). Baugh et al. [31] combined Poisson-image-blending-based synthetic tasks with deformation and intensity-based tasks to form a cross-validation framework, where models were trained to segment one set of synthetic anomaly tasks whilst early-stopping using a different distinct set of tasks, with an ensemble of models trained on all possible combinations of tasks used to create a final anomaly map prediction.

#### 1.1.5 Prior work

Baugh et al. [5] condition a diffusion model on a synthetically corrupted version of an input image and train it to restore the image to its original healthy appearance. This combines the interpretability of reconstruction-based methods with the sensitivity of synthetic anomaly training using the tasks from Baugh et al. [31]. Concurrently, Naval Marimont et al. [32] propose DISYRE, which takes the same approach of training a generative model to reverse synthetic corruptions, but replaces the standard Gaussian noising process with the synthetic anomaly generation process itself, using a cold diffusion framework in which the model learns to invert progressively more severe corruptions back to their healthy originals.

## 2 Materials and methods

Our generative framework operates in the latent space of a pretrained VAE [7]. Given an input image x, the encoder E maps it to a latent representation z=E(x), and the decoder D reconstructs an image from a latent code. We denote the reconstructed image as $x^{\prime }=D(E(x))$, explicitly acknowledging that $x^{\prime }\neq x$ due to the lossy compression of the autoencoder. This distinction becomes important when computing anomaly maps (Section 2.4).

Working in latent space allows the generative model to operate on a more compact and structured representation compared to pixel space, improving computational efficiency and stability.

To show our method’s progression from previous work, we consider two generative formulations for modelling the distribution of healthy anatomy in this latent space: Denoising Diffusion Probabilistic Models (Section 2.1) and Rectified Flow (Section 2.2). Both approaches learn to transform samples from a noise distribution into realistic data, but differ in how this transformation is parameterised and what sort of path the samples take. DDPMs use a discrete sequence of denoising steps, while Rectified Flow models a continuous transformation from noise to data. Note that the two formulations use opposite time conventions: in diffusion, t is defined by the forward corruption process and progresses from the clean latent at t=0 toward noise at t=T, whereas in rectified flow t∈[0,1] indexes the generative trajectory from noise at t=0 to the clean latent at t=1.

### 2.1 Denoising diffusion probabilistic models

A DDPM [33] defines a generative process by progressively corrupting a clean latent $z_{0}$ with Gaussian noise and learning to reverse this corruption. This latent diffusion formulation has been shown to be effective for 3D medical image synthesis in MAISI [11].

A noisy latent at timestep t can be sampled directly as:

$$z_{t}=\sqrt{{\bar{\alpha }}_{t}}\;z_{0}+\sqrt{1-{\bar{\alpha }}_{t}}\;\epsilon ,$$ (1)

where ϵ∼N(0,I) and ${\bar{\alpha }}_{t}$ controls the noise level. As t increases, the latent becomes increasingly dominated by noise.

A neural network $\epsilon_{\theta }(z_{t},t)$ is trained to predict the noise component ϵ from the noisy latent. The model is trained using the standard ϵ-prediction objective:

$$L_{\text{DDPM}}=E_{z_{t},\epsilon },t\left[{\left|\epsilon -\epsilon_{\theta }(z_{t},t)\right|}^{2}\right].$$ (2)

At inference, generation starts from a noise sample and iteratively applies the learned denoising model to produce a latent ${\hat{z}}_{0}$, which can then be decoded to an image $\hat{x}=D({\hat{z}}_{0})$.

### 2.2 Rectified flow models

Rectified Flow [34–36] provides an alternative formulation in which the transformation from noise to data is modelled as a continuous deterministic process. Instead of predicting noise at discrete timesteps, the model learns a velocity field that describes how a sample evolves over time.

Starting from $z_{0}∼N(0,I)$, the latent evolves according to the ordinary differential equation:

$$\frac{dz_{t}}{dt}=v_{\theta }(z_{t},t),\;t\in [0,1],$$ (3)

where t=0 corresponds to noise and t=1 corresponds to data.

Intermediate states are defined by linear interpolation between a noise sample $z_{0}$ and a data latent $z_{1}=E(x)$:

$$z_{t}=(1-t)z_{0}+tz_{1}.$$ (4)

The model is trained to predict the displacement between these endpoints:

$$L_{\text{\textbackslash{},flow}}=E_{z_{0},z_{1},t}\left[\|v_{\theta }(z_{t},t)-(z_{1}-z_{0}){\|}_{2}^{2}\right].$$ (5)

At inference, the learned velocity field is integrated from t=0 to t=1 to obtain a latent ${\hat{z}}_{1}$, which is decoded to an image $\hat{x}=D({\hat{z}}_{1})$.

### 2.3 Self-supervised anomaly detection

**Figure 2:**
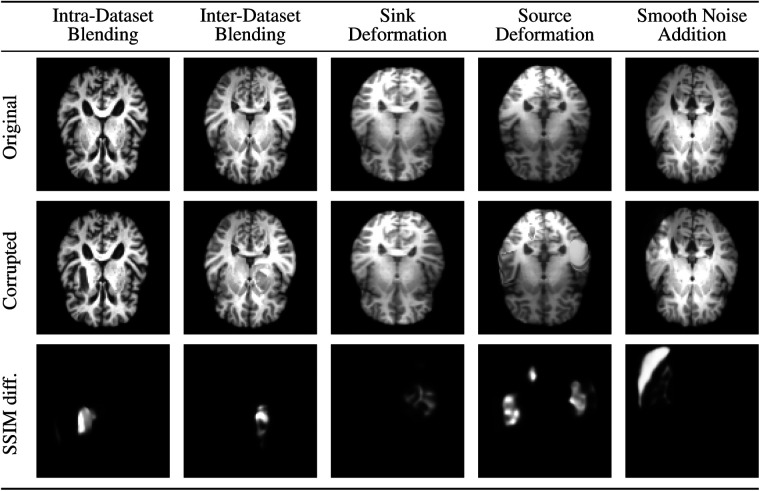
Examples of each of the synthetic anomaly tasks applied to T1-weighted brain MRI. Each task is applied to the full 3D volume up to 3 times although we only visualise the central axial slice here.

Self-supervised anomaly detection generally involves training a model to directly localise synthetic anomalies introduced into otherwise normal images, so the model can then directly identify real anomalies at test time. It is worth noting that the aim of using synthetic anomalies for training is to use such a broad range of highly varied synthetic anomalies that the model is encouraged to learn the characteristics of the healthy data. We specifically do not aim to simulate specific pathologies, as if that were the case, the models would fall into the same trap as supervised models; by tailoring the synthetic anomalies to the target anomalies they cease to provide an accurate measure of how the models would generalise to unknown anomalies in the wild. Keeping with these principles, we follow the formulation of Baugh et al. [5], in which a model is trained to correct a wide range of anomalies rather than localise them (see Section 2.4). We use the 3D synthetic tasks from Baugh et al. [31], with the same adaptation of Baugh et al. [5] in using layered fractal noise instead of a uniform intensity for the intensity addition. This makes the set of synthetic tasks (Figure 2):

1. **Intra-dataset Blending:** patches from the same dataset are Poisson blended into the current image. Uses the D-dimensional Poisson image editing method proposed in Baugh et al. [31].
2. **Inter-dataset Blending:** as above, but samples are taken from other datasets; brain MRI models use patches from AADD CT images and vice versa.
3. **Sink Deformation:** voxels are pulled towards a randomly selected point within the patch.
4. **Source Deformation:** voxels are pushed away from a randomly selected point within the patch.
5. **Smooth Noise Addition:** a random number of fractal noise samples at different scales are either added or subtracted from the region, with the edges being smoothed to integrate into the surrounding image.

For each corrupted train sample, the chosen task is applied up to 4 times. Each application uses an ellipsoid or cuboid mask and selects a random location such that at least 50% of the mask intersects with the foreground of the image. To create latent synthetic anomalies (ImgCondRFLowLT${}_{ens.}$ in Tables 1 and 3), we apply the same anomaly creation pipeline to the latent encodings with minimal modifications: we use latent encodings of images as patches for blending, and we remove the foreground restriction (as the latent encoding of an image has no clear foreground).

**Table 1:** Quantitative detection and localisation results on the BraTS-T2, BraTS-T1 and ATLASv2 brain MRI datasets.

			BraTS-T2				BraTS-T1				ATLASv2			
			Slice-level		Pixel-level		Slice-level		Pixel-level		Slice-level		Pixel-level	
Paradigm	Method	Dim.	AP_i_	AUC_i_	AP_p_	⌈Dsc⌉	AP_i_	AUC_i_	AP_p_	⌈Dsc⌉	AP_i_	AUC_i_	AP_p_	⌈Dsc⌉
	Random		48.1	50.0	2.20	4.30	48.0	50.0	2.19	4.29	29.4	50.0	0.528	1.05
	ForegroundPred		81.0	85.4	6.4	12.1	81.0	85.4	6.5	12.1	60.6	81.8	1.6	3.1
**IR**	VAE [7]	2D	53.8	60.3	20.1	28.1	63.2	72.6	14.6	21.6	38.0	65.9	5.7	11.7
	r-VAE [8]	2D	65.2	73.0	31.2	41.1	59.3	68.8	21.4	29.7	36.5	65.3	8.8	17.5
	f-AnoGAN [10]	2D	72.2	79.3	6.9	13.0	44.8	43.4	8.5	17.3	22.7	35.2	2.1	5.2
	H-TAE-S [9]	2D	44.6	48.3	11.1	18.2	45.9	43.2	11.7	21.1	23.6	39.6	2.5	4.7
	MAISI-VAE [6]	3D	51.2	57.1	5.8	13.0	56.0	61.3	10.3	17.6	32.1	56.4	3.2	6.6
	MAISI-VAE${}_{\;ft}$ [6]	3D	50.2	56.1	5.9	13.0	55.7	61.1	10.8	18.3	32.0	56.3	3.2	6.6
	MAISI-RFlow [6]	3D	55.2	60.2	7.9	14.0	54.3	61.8	5.4	13.0	34.0	57.0	1.3	3.5
	MAISI-RFlow${}_{\;ft}$ [6]	3D	73.2	77.3	17.3	24.8	76.2	80.2	25.4	32.4	64.8	80.3	25.7	31.5
**FM**	FAE [16]	2D	84.6	84.9	45.4	47.6	83.7	84.3	38.3	42.3	59.9	76.2	23.6	32.4
	PaDiM [18]	2D	74.4	73.9	41.4	43.4	61.7	66.2	21.3	29.1	42.7	64.4	11.5	21.8
	CFLOW-AD [19]	2D	74.5	74.1	34.8	39.3	65.1	70.1	17.0	25.6	48.9	65.2	13.5	23.7
	RD [20]	2D	87.1	86.5	53.8	54.2	80.0	82.4	38.3	43.2	65.3	80.7	27.5	39.0
**AB**	AMCons [23]	2D	46.3	45.9	19.3	25.6	48.8	45.6	6.7	12.1	24.6	41.4	1.1	3.1
	ExpVAE [22]	2D	72.8	75.2	29.0	36.6	63.2	71.5	5.8	12.3	30.8	55.4	1.1	3.1
**S-S**	PII [28]	2D	57.2	63.3	17.4	25.4	54.7	63.3	10.5	20.1	44.1	69.1	4.6	10.3
	DAE [25]	2D	53.0	59.8	27.3	39.5	58.0	64.6	33.2	40.7	30.9	55.8	15.4	24.8
	CutPaste [26]	2D	77.2	78.1	13.6	20.2	61.9	69.4	7.0	12.9	51.4	69.6	6.4	12.8
	ImgCondDDPM${}_{ens.}$ [5]	2D	**92.8**	91.4	62.5	63.0	86.2	86.2	47.8	**53.1**	**75.3**	85.4	44.8	53.0
	ImgCondRFlow${}_{ens.}$	3D	90.9	91.5	58.7	59.5	84.6	86.6	41.6	49.2	74.2	88.5	47.3	52.9
	ImgCondRFlowLT${}_{ens.}$	3D	91.1	90.9	62.5	59.6	**88.7**	**89.1**	**53.6**	52.9	74.2	86.7	37.9	47.8
	ImgCondRFlowLTAvg${}_{ens.}$	3D	91.7	**92.0**	**68.3**	**63.6**	86.1	87.6	53.1	52.9	74.9	**88.8**	**48.6**	**54.2**

We compare against image-reconstruction (**IR**), feature-modelling (**FM**), attention-based (**AB**), and self-supervised anomaly detection (**S-S**) methods.

Best scores are bold, second best are underlined.

### 2.4 Image conditioning for anomaly restoration

Following a similar intuition to that of Baugh et al. [5], we train our models to denoise healthy regions whilst restoring synthetically corrupted regions to their healthy counterparts. Unlike Baugh et al. [5] which operates on images, our model takes in two latent encodings obtained using MAISI’s pretrained VAE encoder: the primary input is the healthy but partially noised $z_{t}$ and the conditioning sample is the encoding of the same image after being corrupted by a synthetic anomaly $\tilde{z}$. Synthetic anomalies are generated in the same fashion as Baugh et al. [5], with each model training on three out of the five tasks. We then train 10 different U-Nets (one for each distinct combination of synthetic anomaly tasks). Figure 1 demonstrates the process of training one of these U-Nets.

**Figure 3:**
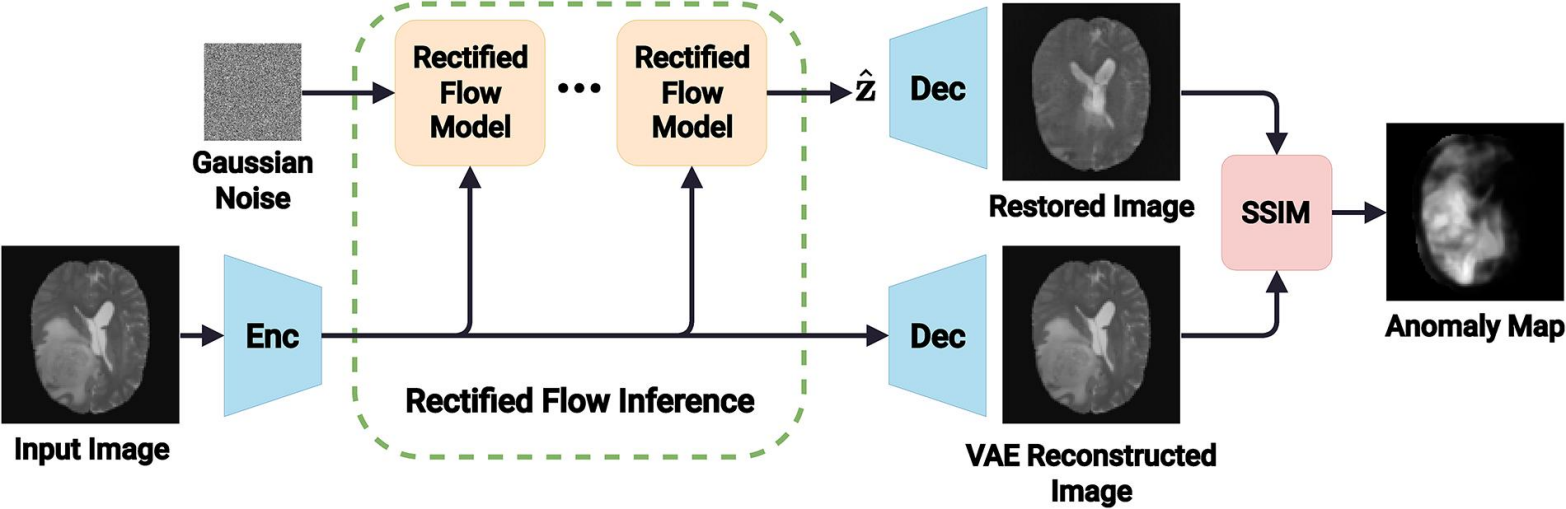
Our inference pipeline. For each model in the ensemble an anomaly map is computed as the SSIM between the restored image and the reconstruction of the input image. The final anomaly prediction is obtained by taking the mean across all anomaly maps.

For inference, given an input image x, each model produces a pseudo-healthy restoration $\hat{x}$ by conditioning its generative process on the encoding of the input image z=E(x). Initially, we followed the standard reconstruction-based anomaly detection setup of using the SSIM to compare the restored image $\hat{x}$ to the original image x in order to create an anomaly map, but we found that this resulted in many false positives across the image due to the compression of the pretrained VAE. To mitigate this, we instead compared the restored image $\hat{x}$ to the input image after being reconstructed with the VAE $x^{\prime }=D(E(x))$. This way, both $\hat{x}$ and $x^{\prime }$ include the changes due to the VAE compression, but differ in that the latent of $\hat{x}$ was restored into the healthy distribution by the rectified flow model. Figure 3 illustrates the full pipeline for an individual U-Net. The anomaly maps from each model are then averaged to get a final prediction. We follow Lagogiannis et al. [21] in zeroing the anomaly score for all background pixels for the brain MRI images (including the AADD Brain dataset). The slice-wise anomaly score is the mean of the scores for that slice.

We found that, despite it not being used in the backpropagation process, applying the frozen VAE encoder was the principal bottleneck of our training pipeline, with it occupying ∼60% of total training time even after we used caching so that the encoder was only being applied to the dynamically corrupted images. To address this, we propose an alternative approach in which the latent encoding of a healthy image is directly perturbed to produce an anomalous encoding. This reformulation allows the VAE encoding of the healthy image to be cached ahead of time, and then used for both the Rectified Flow scheduler and synthetic anomaly generation. As an added bonus, we found this to allow for higher resolution images, specifically on the brain MRI datasets we could take the native (160,192,160) sized images as input.

### 2.5 Datasets

For training our primary brain models, we train one on T1-weighted MR images and another on T2-weighted MR images. To do this, we have three datasets consisting of only healthy samples; the CamCan [37] dataset containing 653 images of both T1 and T2 weighting, the HCP [38] dataset containing 1,071 images of both T1 and T2 weighting, and the IXI [39] dataset which contains 1,159 MR images, although we only use the 581 T1-weighted images and 577 T2-weighted images which have a corresponding T1-weighted image as our preprocessing aligns all images to a T1-weighted atlas. For evaluation, we use the BraTS 2020 [40–42] training data together with the ATLASv2 [43] dataset. BraTS’20 consists of 369 samples, with large lesions that present as hyperintense in T2-weighted scans and lower contrast in T1-weighted scans, making them harder to identify in the T1 setting despite the same size of lesions. We denote the BraTS’20 T2-weighted subset as BraTS-T2 and likewise the T1-weighted subset as BraTS-T1. The ATLASv2 dataset contains 655 T1-weighted scans of stroke patients, with small and hypo-intense lesions appearing in the scans, resulting in a low proportion of 30% anomalous axial slices.

We also evaluate our model on the Advancing Anomaly Detection Dataset (AADD) [44], a benchmark designed to address a common concern in anomaly detection research, that methods trained with synthetic anomalies cannot be tested on datasets with synthetic anomalies, even those distinct from the training set, as they may exploit shared synthetic artefacts rather than learning to detect genuinely meaningful deviations. To mitigate this, AADD applies synthetic transformations to both normal and anomalous classes, reducing the domain shift that arises when only the test anomalous samples are altered and thus preventing the shortcut of learning to identify synthetic artefacts. AADD consists of 2 sub-datasets, AADD Brain and AADD Abdominal, which are both derived from the training data of the MOOD challenge [45]. In AADD Brain, normal samples have a band of spatial frequencies removed and are rotated about a random axis by a moderate angle; anomalous samples differ from this baseline in four possible ways: over-rotation, under-rotation, the restoration of the removed frequencies globally, or the localised addition of spatial frequencies within a specific region. The first three classes apply global transformations, producing global rather than localised anomalies. In AADD Abdominal, a small synthetic organ is inserted into all samples with anomalous samples exhibiting one of four alterations to this organ, namely added texture, a morphological split into two smaller structures, displacement to an abnormal location, or complete absence. The synthetic organ is spatially small, resulting in a high degree of class imbalance at both the volume and pixel levels, making it a challenge for detection and localisation.

#### 2.5.1 Data preprocessing

The first stage of the data preprocessing is to compute atlas-space images for all MRI volumes and lesion maps if present. For IXI dataset only, this stage begins with an affine alignment of T2 to T1 volumes using ANTsPy. Then, brain extraction is performed via HD-BET on T1 images for applicable datasets (CamCAN, ATLAS, IXI), and the resulting masks are propagated to corresponding T2 volumes. BraTS and HCP are already skull-stripped and thus do not require this brain extraction step. We register all MRI volumes and available lesion masks to SRI24 atlas using ANTsPy affine transformation, resulting in atlas-space images with shape (240,240,155) for the next stage.

The second stage commences with cropping each image to a minimal brain bounding box and zero-padding to a uniform dimension of (160,192,160). Following Zhao et al. [6], the voxel intensities are normalised to a maximum of 1 based on the 99.5th percentile of the whole volume. For 2D baseline models, only non-empty axial slices are kept, and each slice is first downsampled to (80,96) and then zero-padded to (96,96) to accommodate standard square-input requirements. In contrast, 3D models use full volumes resized to (80,96,80). And to ensure an equitable comparison across dimensions, background-only axial slices are also discarded from 3D anomaly maps prior to metric computation, and identical spatial padding is applied.

AADD datasets are pre-normalised to a range [0,1], and thus separate preprocessing is applied. Since data preprocessing in Zhao et al. [6] normalises CT volumes from a fixed HU range [−1,000,1,000] to [0,1], which matches the range of AADD Abdominal images, we only resize each volume to (28,128,128) shape for AADD Abdominal images. For AADD brain subsets evaluated with MAISI-VAE and MAISI-RFlow methods [6], we also apply the 99.5th percentile normalisation to maintain latent alignment with pretrained model representations before resizing to (128,128,128).

### 2.6 Implementation details

All models are trained on either A40 or A100 40GB GPUs. We use the same baseline implementations as Baugh et al. [5], only changing the data preprocessing pipeline as described in Section 2.5, with the singular exception of fixing a bug in the method proposed by Baugh et al. [5] where the attention mechanism was being applied at the higher resolutions within the diffusion U-Net instead of the lower resolutions. This resulted in a considerable increase in performance, surpassing all its original baselines when it was previously behind some methods when evaluated on T1-weighted brain MRI.

We also use the MAISI VAE (MAISI-VAE) and rectified flow models (MAISI-RFlow) for anomaly detection, providing 3D model comparisons. For completeness we compare both the base pretrained models and the result of fine-tuning them on the normal data for the current task (MAISI-[VAE|RFlow]${}_{\;ft}$). The MAISI-VAE models identify anomalies by reconstructing the image and comparing the result to the original, while MAISI-RFlow restores the image by partially noising and denoising its latent space representation, and comparing the decoded result ($\hat{x}$) to the VAE reconstruction of the original ($x^{\prime }$). As expected the base models perform very badly due to being trained on a wide range of images, so they are able to reconstruct both healthy and unhealthy regions.

As a naïve baseline, we implement a fixed model “ForegroundPred” which predicts 1 for all pixels greater than the minimum of the slice (i.e., the foreground), and takes the average pixel-wise prediction as the slice-wise score. In this way it simply predicts the largest slices, assuming that more pixels means a higher chance of containing an anomaly.

## 3 Results

**Figure 4:**
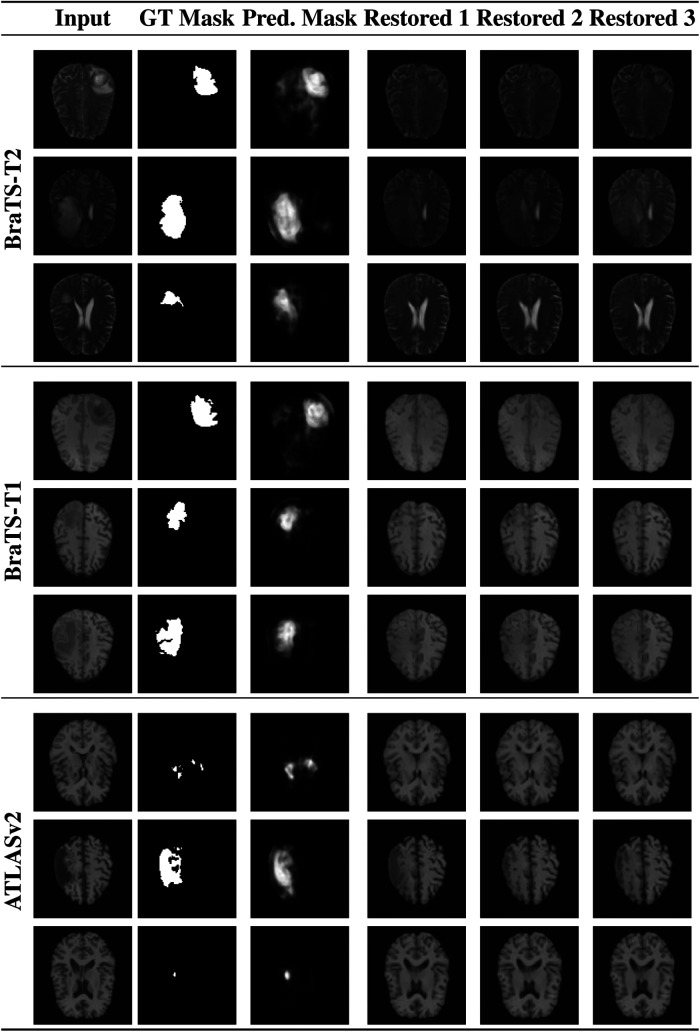
Qualitative results across three datasets. Each row shows one sample: the original input image, the ground truth anomaly segmentation mask, the ImgCondRFlowLTAvg${}_{ens.}$ model’s predicted anomaly map, and three examples of pseudo-healthy restorations.

We evaluate the results of our model trained with anomaly imputation in the pixel space (ImgCondRFlow${}_{ens.}$), anomaly imputation in the latent space (ImgCondRFlowLT${}_{ens.}$), and the average of the two (ImgCondRFlowLTAvg${}_{ens.}$). Together our models set a new state of the art, with strong performance across BraTS-T2, BraTS-T1 and ATLASv2 (Table 1). Where other methods are capable of good localisation on the more obvious hyperintense anomalies of BraTS-T2 but then perform worse on the lower-contrast ones, our models are able to locate even challenging anomalies as shown by ImgCondRFlowLTAvg${}_{ens.}$ reaching a pixel-level average precision of 68.3, 53.1 and 48.6 on the BraTS-T2, BraTS-T1, and ATLASv2 datasets respectively. This high, consistent performance shows that the models trained with pixel-space anomaly imputation (ImgCondRFlow${}_{ens.}$) and those trained with latent-space anomaly imputation (ImgCondRFlowLT${}_{ens.}$) learn complementary features of the normal distribution, leading to strong ensembled performance. The only baseline with comparable performance is ImgCondDDPM${}_{ens.}$ [5], which is largely due to a bug fix we identified, and is ∼10 times slower than the 3D models at the same resolution (ImgCondRFlow${}_{ens.}$). Figure 4 shows qualitative results, demonstrating our model’s ability to localise anomalies of different sizes, contrasts and topologies. In the third row of Figure 4 we see a limitation of evaluating on pathology-centric datasets such as these; although the upper left ventricle is deformed by the nearby pathology it is not included in the ground truth segmentation as that specifically segments the tumour. Many of our models corrected both the tumour and the ventricle, but due to the labelling the ventricle restoration actually harms the performance metrics. To remedy this in the future, it would be good to collect anomaly detection datasets annotated for all forms of anomaly, including the pathologies and the deformations they cause.

One interesting observation is that although both ImgCondRFlow${}_{ens.}$ and ImgCondRFlowLT${}_{ens.}$ compute the anomaly map using the same method, ImgCondRFlowLT${}_{ens.}$ seems notably better at localising the low-contrast anomalies of BraTS-T1 (AP_p_: 53.6>41.6, ⌈Dsc⌉: 52.9>49.2). This shows that, although synthetic anomalies created in the latent space don’t directly correspond to an anomaly in pixel-space, they are still useful for learning the features of the normal distribution.

For the AADD datasets, we only train and ensemble four folds as a trade-off between performance and computational efficiency, motivated by Section 3.2. Despite this we achieve strong performance, while many other methods struggle to improve upon the naïve baselines (Table 2). On the AADD Brain dataset, our ImgCondRFlow${}_{ens.}$ beats the average precision score of the second-best methods by 12.5 at a slice level and 13.7 at a pixel level. This could be because three of the four anomalous classes are global, so where 2D methods process each slice independently are unable to see the full picture, our 3D model can identify the change. On the AADD Abdominal dataset, our model achieves strong slice-level performance but strangely falls far behind its 2D predecessor at a pixel level. This may be because the dataset centres on a small synthetic organ that never appeared in the pretrained VAE’s training data. The VAE’s latent space is therefore unlikely to effectively encode that organ, which limits the pipeline’s ability to flag anomalies in that organ.

**Table 2:** Quantitative detection and localisation results on the AADD datasets.

			AADD brain				AADD abdominal			
			Slice-level		Pixel-level		Slice-level		Pixel-level	
Paradigm	Method	Dim.	AP_s_	AUC_s_	AP_p_	⌈Dsc⌉	AP_s_	AUC_s_	AP_p_	⌈Dsc⌉
	Random		41.9	50.0	11.8	21.1	4.38	50.0	0.033	0.066
	ForegroundPred		43.7	52.2	39.7	56.8	3.39	40.9	0.038	0.075
**IR**	VAE [7]	2D	42.4	51.1	43.7	56.9	4.04	48.9	0.056	0.168
	r-VAE [8]	2D	46.7	56.7	43.8	57.0	4.28	43.7	0.030	0.066
	f-AnoGAN [10]	2D	48.6	55.7	40.5	56.8	4.68	51.4	0.055	0.138
	H-TAE-S [9]	2D	43.2	53.1	40.1	56.8	6.35	60.3	0.020	0.066
	MAISI-VAE [6]	3D	41.1	49.3	39.4	56.8	7.33	68.3	0.029	0.082
	MAISI-VAE${}_{\;ft}$ [6]	3D	47.1	53.9	41.6	56.8	7.73	71.1	0.031	0.083
	MAISI-RFlow [6]	3D	41.2	49.0	39.2	56.8	5.39	53.8	0.066	0.145
	MAISI-RFlow${}_{\;ft}$ [6]	3D	37.6	43.1	41.1	56.9	4.46	50.8	0.051	0.125
**FM**	FAE [16]	2D	56.1	61.2	50.5	57.1	3.91	44.9	0.090	0.210
	PaDiM [18]	2D	49.8	56.0	45.3	57.0	4.11	47.8	0.078	0.181
	CFLOW-AD [19]	2D	49.1	55.8	43.5	56.8	3.49	40.2	0.099	0.275
	RD [20]	2D	51.5	59.9	47.3	57.1	5.00	51.7	0.154	0.435
**AB**	ExpVAE [22]	2D	44.8	52.9	36.3	56.8	3.74	47.0	0.107	0.236
	AMCons [23]	2D	42.9	51.6	41.3	57.1	3.08	35.0	0.095	0.433
**S-S**	PII [28]	2D	45.5	51.9	37.8	56.8	3.97	48.4	0.151	0.925
	DAE [25]	2D	41.6	51.1	39.0	56.8	3.72	44.8	0.044	0.096
	CutPaste [26]	2D	52.2	59.6	40.9	56.8	4.07	47.6	0.048	0.130
	ImgCondDDPM${}_{ens.}$ [5]	2D	62.8	64.2	48.0	56.8	**11.98**	69.4	**1.454**	**8.189**
	ImgCondRFlow${}_{ens.}$	3D	**75.3**	**73.8**	**64.2**	**61.3**	10.21	**76.4**	0.114	0.417

We compare against image-reconstruction (**IR**), feature-modelling (**FM**), attention-based (**AB**), and self-supervised anomaly detection (**S-S**) methods.

Best scores are bold, second best are underlined.

### 3.1 Model components ablation

**Figure 5:**
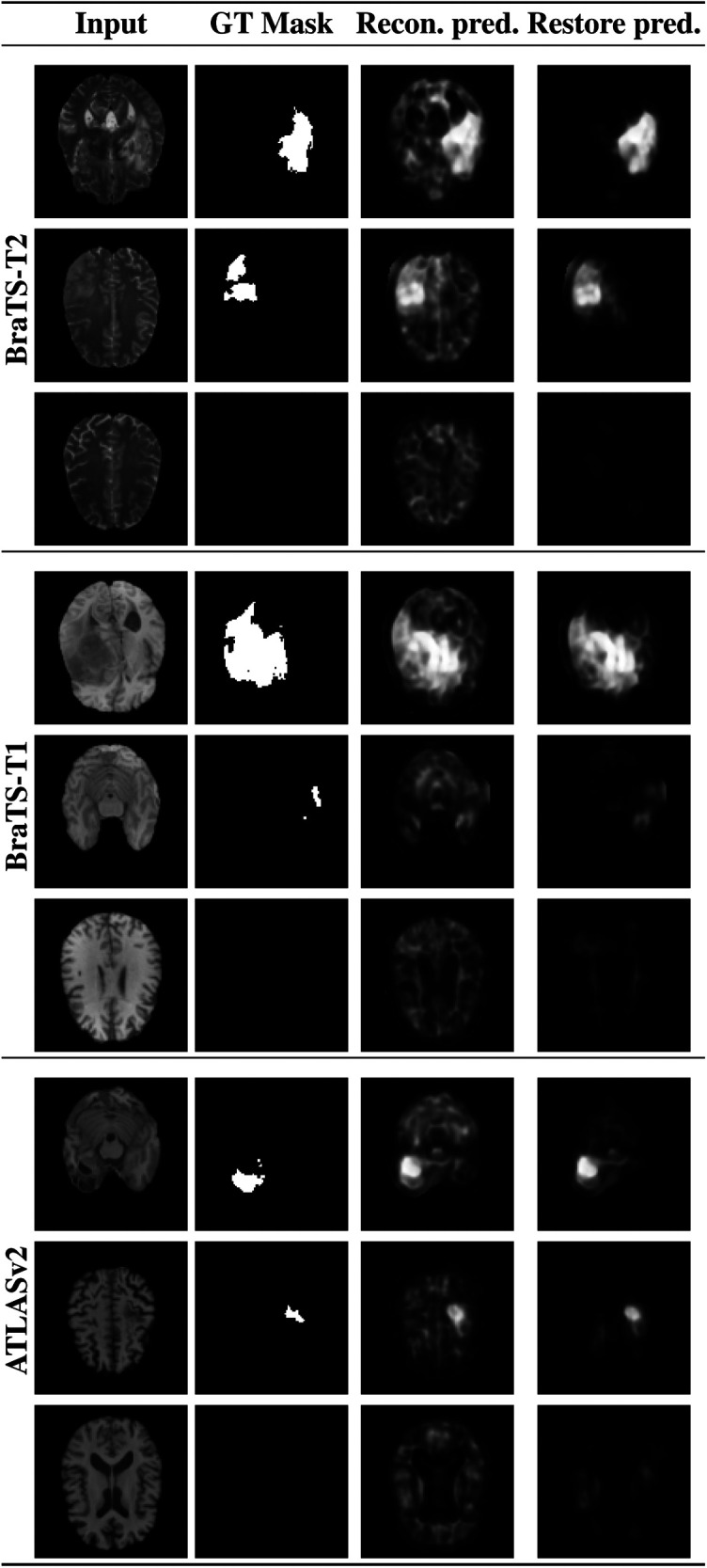
Qualitative comparison of reconstruction-based vs. restoration-based anomaly maps. Although predictions use the same model, “Recon. Pred.” compares the pseudo-healthy restored image to the original input image (x) while “Restore Pred.” compares it to the VAE reconstruction of the original ($x^{\prime }=D(E(x))$. “Recon. Pred” has more false positive predictions due to small compression artefacts introduced by the VAE, which can be avoided by comparing with $x^{\prime }$ instead.

Table 3 traces the progression of each design choice. Starting from the pretrained MAISI-RFlow model without any fine-tuning (first group in Table 3), we observe modest slice-level detection but also performance degradation as the noise level decreases. Furthermore, it shows almost no pixel-level localisation, as the resulting reconstruction errors are spread uniformly across the image rather than concentrated at anomalous regions. This is likely due to the fact that the VAE draws from the mean of its wide training distribution and effectively reconstructs a plausible silhouette rather than faithfully preserving the input. Although fine-tuning MAISI-RFlow on the training data (MAISI${}_{\;ft}$) substantially improves localisation, the choice of noise level reveals a clear trade-off. Higher starting noise ($T_{start}=0.4$) benefits the detection of the prominent hyperintense lesions in BraTS-T2, whilst lower noise ($T_{start}=0.6$) is preferable for the lower-contrast BraTS-T1 anomalies and the smaller lesions of ATLASv2, since less aggressive noising better preserves fine structural detail. Switching from a pixel-wise absolute difference ($\left|x-\hat{x}\right|$) to the structural SSIM-based anomaly function consistently improves performance. And using the VAE reconstruction of the original ($x^{\prime }$) as the comparison reference rather than the raw input x yields a further gain by cancelling the false positives introduced by lossy VAE compression (qualitative examples shown in Figure 5). Adding image-conditioned training brings by far the largest improvement, with ImgCondRFlow${}_{ens.}$ using $\text{DSSIM}(x,\hat{x})$ already surpassing the best MAISI-RFlow${}_{\;ft}$ by over 30 points in pixel-level AP_p_ on BraTS-T2 (22.3 → 53.9), over 15 points on BraTS-T1 (25.4 → 41.2), and nearly 20 points on ATLASv2 (25.7 → 45.0). Combining this with the restoration-change comparison ($\text{DSSIM}(x^{\prime },\hat{x})$) improves performance further, particularly on ATLASv2.

**Table 3:** Ablation study tracing the progression of each design choice on the BraTS-T2, BraTS-T1 and ATLASv2 brain MRI datasets.

			BraTS-T2				BraTS-T1				ATLASv2			
Method			Slice-level		Pixel-level		Slice-level		Pixel-level		Slice-level		Pixel-level	
Model	Anom. fn.	$T_{start}$	AP_i_	AUC_i_	AP_p_	⌈Dsc⌉	AP_i_	AUC_i_	AP_p_	⌈Dsc⌉	AP_i_	AUC_i_	AP_p_	⌈Dsc⌉
MAISI-RFlow	$\left|x-\hat{x}\right|$	0.2	55.2	60.2	7.9	14.0	54.3	61.8	5.4	13.0	34.0	57.0	1.3	3.5
		0.4	48.3	51.6	6.5	13.0	53.4	58.8	5.5	13.0	30.5	51.1	1.6	3.5
		0.6	45.7	48.2	6.8	13.1	54.9	59.7	5.6	13.0	29.9	49.3	1.7	3.5
		0.8	45.4	47.2	6.0	13.0	54.9	59.5	5.6	13.0	30.7	49.5	1.8	3.5
MAISI-RFlow${}_{\;ft}$	$\left|x-\hat{x}\right|$	0.2	47.9	51.9	22.3	32.5	54.9	61.6	8.6	14.9	30.3	50.8	8.5	16.9
		0.4	45.8	48.3	21.6	31.1	54.7	60.8	8.9	15.2	31.8	53.0	11.3	20.8
		0.6	44.9	46.7	13.5	22.5	54.8	60.0	9.7	16.6	32.6	54.3	11.9	21.5
		0.8	45.4	47.0	8.6	16.0	54.8	59.6	5.9	13.0	31.4	50.6	2.3	4.8
	$DSSIM(x,\hat{x})$	0.2	51.0	54.7	16.3	25.4	52.6	57.5	13.9	22.6	33.4	56.3	6.5	13.7
		0.4	60.2	68.6	21.4	29.6	61.6	70.3	20.3	28.5	41.1	68.1	17.2	27.1
		0.6	70.6	74.7	14.0	21.1	74.9	77.9	22.6	29.5	51.5	72.4	16.7	23.1
		0.8	52.9	59.5	6.6	13.0	56.4	62.0	12.2	20.3	34.0	59.2	4.2	8.3
	$DSSIM(x^{\prime },\hat{x})$	0.2	51.1	54.8	16.3	25.4	52.4	57.2	14.0	22.6	33.4	56.2	6.6	13.7
		0.4	61.6	70.2	22.1	30.5	62.6	71.5	20.7	29.0	42.8	70.1	18.4	28.4
		0.6	73.2	77.3	17.3	24.8	76.2	80.2	25.4	32.4	64.8	80.3	25.7	31.5
		0.8	63.3	69.5	9.5	14.9	65.1	72.4	14.9	23.0	48.4	72.0	7.0	12.2
ImgCondRFlow${}_{ens.}$	$\left|x-\hat{x}\right|$	0.0	91.3	91.3	45.1	48.7	84.2	87.6	17.1	26.0	72.0	86.6	28.0	38.9
	$DSSIM(x,\hat{x})$	0.0	91.3	91.4	53.9	56.5	87.6	89.0	41.2	49.3	71.8	86.5	45.0	51.9
	$DSSIM(x^{\prime },\hat{x})$	0.0	90.9	91.5	58.7	59.5	84.6	86.6	41.6	49.2	74.2	88.5	47.3	52.9
	$DSSIM(z,\hat{z})$	0.0	87.1	88.7	46.5	54.0	80.5	83.3	37.3	46.6	71.5	87.4	39.9	45.7
	$DSSIM(x^{\prime },x)+DSSIM(z,\hat{z})$	0.0	89.3	90.3	56.5	59.6	82.7	85.1	41.4	50.2	73.1	88.2	45.9	51.8
	$DSSIM(x^{\prime },x)\times DSSIM(z,\hat{z})$	0.0	89.3	90.2	53.7	57.7	82.6	84.8	40.8	49.9	73.4	87.7	44.0	50.6
ImgCondRFlowLT${}_{ens.}$	$DSSIM(x^{\prime },\hat{x})$	0.0	91.1	90.9	62.5	59.6	**88.7**	**89.1**	**53.6**	**52.9**	74.2	86.7	37.9	47.8
ImgCondRFlowLTAvg${}_{ens.}$	$DSSIM(x^{\prime },\hat{x})$	0.0	**91.7**	**92.0**	**68.3**	**63.6**	86.1	87.6	53.1	**52.9**	**74.9**	**88.8**	**48.6**	**54.2**

*T_start_* denotes the noise level at which restoration begins, following the rectified flow convention of *t* = 0 being fully noise.

Anomaly functions are defined as follows: original image x; pseudo-healthy restoration produced by the rectified flow model $\hat{x}$; VAE reconstruction of the original image $x^{\prime }=D(E(x))$; original latent encoding z=E(x); restored latent $\hat{z}$; Structural Dissimilarity Index Measure DSSIM(A,B)=1−SSIM(A,B). Best scores are **bold** and second best are underlined.

We also explored using the change in the latent representation induced by the rectified flow restoration as an anomaly signal, either alone ($\text{DSSIM}(z,\hat{z})$) or combined with the pixel-level SSIM via element-wise averaging or multiplication. While these variants are competitive with most baselines, they fall short of the pixel-level comparison, likely because the 4× spatial downsampling of the VAE latent space produces a very coarse anomaly map when upsampled back to image resolution.

We note that training with latent-space anomaly imputation (ImgCondRFlowLT${}_{ens.}$) and averaging the pixel-space and latent-space ensemble predictions (ImgCondRFlowLTAvg${}_{ens.}$) yield complementary benefits, as discussed in detail in the main results.

### 3.2 Ensemble size ablation

**Figure 6:**
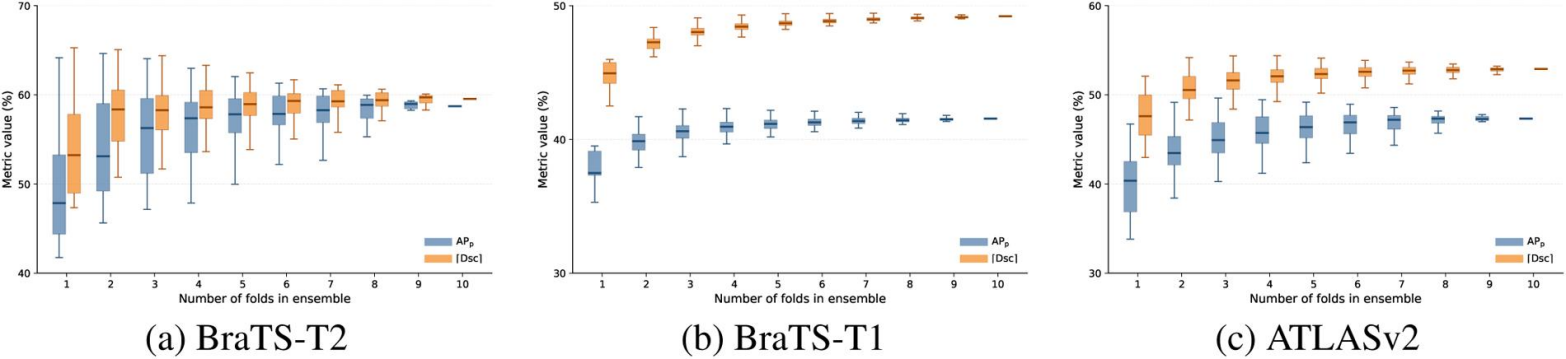
Effect of ensemble size on anomaly segmentation performance using BraTS and ATLASv2. For each ensemble size k∈{1,…,10}, we evaluate all (10k) fold combinations and report the distribution of AP_p_ (blue) and ⌈Dsc⌉ (orange) as box-and-whisker plots. Boxes indicate the interquartile range (IQR), centre lines indicate medians, and whiskers show the full observed range across combinations. **(a)** BraTS-T2, **(b)** BraTS-T1, **(c)** ATLASv2.

As training 10 models per ensemble requires a lot of computational resources, we investigated how performance varies when ensembling different-sized subsets of the ensemble on the primary brain data (Figure 6), focusing on the localisation metrics as they vary more across models. As expected, increasing the size of the ensemble improves the median performance and decreases the variance of the performance. Most of the gains in median performance are acquired after ensembling four or five models, with further models giving consistent but small performance boosts. That four to five ensemble mark is also when the maximum ensemble performance starts to decrease for BraTS-T1 and ATLASv2. However, we see that for BraTS-T2 individual folds are capable of the highest performance when applied alone, although the variance across folds is very high. This suggests that certain task combinations result in a model which is well aligned for BraTS-T2’s hyperintense anomalies, while others are far less aligned, which makes intuitive sense as the dataset blending and smooth noise addition tasks are likely to produce hyperintense synthetic anomalies while the deformation-based tasks are not.

## 4 Discussion

In this work we propose using image-conditioning with latent rectified flow models to perform 3D medical anomaly detection and localisation. Our use of 3D context and pretrained latent space allows for realistic, pseudo-healthy restoration of input images regardless of anomaly contrast or size, as shown by the strong performance across large, hyperintense anomalies (BraTS-T2), large low-contrast anomalies (BraTS-T1) and small hypointense anomalies (ATLASv2). Despite surpassing all baselines in this area, the challenging AADD dataset shows that there are still performance gains to be sought, in particular for the AADD Abdominal dataset. This dataset is based on data from the MOOD challenge, where the performance on the abdominal task has consistently lagged behind that of the brain task due to the high complexity and variability of abdominal CT data, so it is little surprise that measuring the anomaly detection performance of a small synthetic organ introduced into these images is an immensely challenging task. It may be that for such fine-grained anomalies models require a multi-scale approach, as currently encoding the entire CT volume into a latent space together compresses a large amount of information.

One insight that presents itself as a clear springboard for future work is the use of synthetic anomaly imputation in the latent space. The fact that models trained to restore anomalies created within the latent space were able to better correct the low-contrast anomalies of BraTS-T1 than those models trained with standard pixel-space synthetic anomalies shows how any challenging perturbation, even those which don’t correspond to pixel-space anomalies, can be useful for better learning the normal distribution of data.

Another area for future work is to explore ways to reduce the computational burden required by training a large set of models. Although we showed that good performance was possible with only half the maximum number of folds, it still has a high variance and future work should endeavour to find ways to reduce the number of models required to one.

## Data Availability

Publicly available datasets were analyzed in this study. This data can be found here: Advancing Anomaly Detection Dataset (AADD): https://huggingface.co/datasets/Jemtan11/AADD; CamCan: https://camcan-archive.mrc-cbu.cam.ac.uk/dataaccess/index.php; HCP: https://balsa.wustl.edu/project?project=HCP_YA; IXI: https://brain-development.org/ixi-dataset/; BraTs: https://www.kaggle.com/datasets/awsaf49/brats20-dataset-training-validation; ATLASv2: https://fcon_1000.projects.nitrc.org/indi/retro/atlas.html.
